# Association of Estimated Glomerular Filtration Rate and Urinary Uromodulin Concentrations with Rare Variants Identified by *UMOD* Gene Region Sequencing

**DOI:** 10.1371/journal.pone.0038311

**Published:** 2012-05-31

**Authors:** Anna Köttgen, Qiong Yang, Lawrence C. Shimmin, Adrienne Tin, Céline Schaeffer, Josef Coresh, Xuan Liu, Luca Rampoldi, Shih-Jen Hwang, Eric Boerwinkle, James E. Hixson, W. H. Linda Kao, Caroline S. Fox

**Affiliations:** 1 Department of Epidemiology, Johns Hopkins Bloomberg School of Public Health, Baltimore, Maryland, United States of America; 2 Renal Division, Freiburg University Clinic, Freiburg, Germany; 3 Department of Biostatistics, Boston University School of Public Health, Boston, Massachussets, United States of America; 4 Human Genetics Center, Division of Epidemiology and Disease Control, UT-Houston School of Public Health, Houston, Texas, United States of America; 5 Dulbecco Telethon Institute and Division of Genetics and Cell Biology, San Raffaele Scientific Institute, Milan, Italy; 6 Welch Center for Prevention, Epidemiology and Clinical Research, Johns Hopkins Medical Institutions, Baltimore, Maryland, United States of America; 7 NHLBI's Framingham Heart Study and the Center for Population Studies, Framingham, Massachussets, United States of America; 8 Division of Endocrinology, Brigham and Women's Hospital and Harvard Medical School, Boston, Massachussets, United States of America; Peninsula College of Medicine and Dentistry, United Kingdom

## Abstract

**Background:**

Recent genome-wide association studies (GWAS) have identified common variants in the *UMOD* region associated with kidney function and disease in the general population. To identify novel rare variants as well as common variants that may account for this GWAS signal, the exons and 4 kb upstream region of *UMOD* were sequenced.

**Methodology/Principal Findings:**

Individuals (n = 485) were selected based on presence of the GWAS risk haplotype and chronic kidney disease (CKD) in the ARIC Study and on the extremes of of the *UMOD* gene product, uromodulin, in urine (Tamm Horsfall protein, THP) in the Framingham Heart Study (FHS). Targeted sequencing was conducted using capillary based Sanger sequencing (3730 DNA Analyzer). Variants were tested for association with THP concentrations and estimated glomerular filtration rate (eGFR), and identified non-synonymous coding variants were genotyped in up to 22,546 follow-up samples. Twenty-four and 63 variants were identified in the 285 ARIC and 200 FHS participants, respectively. In both studies combined, there were 33 common and 54 rare (MAF<0.05) variants. Five non-synonymous rare variants were identified in FHS; borderline enrichment of rare variants was found in the extremes of THP (SKAT p-value = 0.08). Only V458L was associated with THP in the FHS general-population validation sample (p = 9*10^−3^, n = 2,522), but did not show direction-consistent and significant association with eGFR in both the ARIC (n = 14,635) and FHS (n = 7,520) validation samples. Pooling all non-synonymous rare variants except V458L together showed non-significant associations with THP and eGFR in the FHS validation sample. Functional studies of V458L revealed no alternations in protein trafficking.

**Conclusions/Significance:**

Multiple novel rare variants in the *UMOD* region were identified, but none were consistently associated with eGFR in two independent study samples. Only V458L had modest association with THP levels in the general population and thus could not account for the observed GWAS signal.

## Introduction

Chronic kidney disease affects 5–10% of adults worldwide [1], and is associated with an increased risk of cardiovascular morbidity and mortality [2], [3] as well as end-stage renal disease [4], [5]. Using genome-wide association studies (GWAS), we and others have previously identified common variants in the *UMOD* gene region on chromosome 16 that are associated with measures of kidney function, including estimated glomerular filtration rate (eGFR), serum creatinine, and chronic kidney disease (CKD) [6], [7], [8], [9], [10]. The role of the *UMOD* gene in the etiology of kidney diseases is further supported by rare *UMOD* variants causing monogenic forms of cystic kidney disease (MIM #162000, #603860, #609886) [11], [12].

The *UMOD* gene is exclusively transcribed in the kidney and encodes Tamm Horsfall protein, also known as uromodulin, the most abundant protein in the urine of healthy individuals [13]. Both rare monogenic disease-causing mutations and common variants identified by GWAS lead to altered concentrations of urinary uromodulin [10], [14], [15]. Whereas urinary uromodulin concentrations are typically reduced in the urine of individuals with monogenic forms of UMOD-associated disease [16], individuals carrying the CKD risk-increasing variants identified in GWAS have higher amounts of urinary uromodulin concentrations [10], [14], [17].

Genome-wide association studies pinpoint genetic regions rather than causal variants. The objective of this study was therefore to sequence *UMOD* exonic, regulatory and conserved regions in several hundred participants of the Framingham Heart Study (FHS) and the Atherosclerosis Risk in Communities Study (ARIC) selected based on extremes of urinary uromodulin concentrations (FHS) and CKD status and *UMOD* haplotype surrounding the GWAS signal (ARIC). Variants that were associated with kidney phenotypes or resulted in amino acid changes were genotyped in the entire FHS and ARIC studies in order to assess whether any of them may account for the GWAS signal and to confirm and evaluate novel rare variants that may help to explain disease risk.

## Methods

### Ethics Statement

Participants from both the FHS and the ARIC studies provided written informed consent and the institutional review boards from the coordinating institutions approved the study.

### Study samples

The Framingham Heart Study, initiated in 1948, is a community-based longitudinal study. The original cohort of 5,209 participants were randomly enrolled from Framingham, Massachusetts, United States [18]. In 1971, 5,124 children of the original cohort, and children's spouses, were enrolled and referred as the offspring cohort [19]. In 2002, the third generation cohort, 4,095 children of the offspring cohort, was recruited [20]. Most FHS participants are self-identified as Caucasian (white). Among them, genome-wide SNP genotyping of 9,274 participants from all three generations was performed using the Affymetrix 500 K mapping array and the Affymetrix 50 K supplemental array. The study sample for the current study is a subset of individuals attending the Framingham offspring cohort exam 6 (1995–1998) with urinary uromodulin levels (n = 2,948) and with eGFR (n = 3,452) measured, as well as individuals attending the original cohort exam 15 with eGFR levels (n = 2,538) and the third generation with eGFR levels (n = 4,067). Urinary uromodulin concentrations were measured via immunoassay with a bead Luminex platform (Rules Based Medicine, Austin, TX); the inter-assay coefficient of variation (CV) is 5.0% at a mean concentration of 37 ug/ml and 11.4% at a mean concentration of 9.4 ug/ml. Urinary creatinine was measured using the modified Jaffé method by a morning single-void urine sample. Urinary uromodulin was indexed to creatinine as uromodulin-to-creatinine ratio in order to account for differences in urine concentration.

The Atherosclerosis Risk in Communities Study is an ongoing population-based study that enrolled individuals at four US study sites from 1987–89 [21]. Individuals were interviewed and examined at four study visits, approximately every three years. Detailed information on demographics, cardiovascular risk factors and co-morbidities is available and has been described previously [21]. For the current study, data was analyzed from the fourth study visit (1996–98).

In both studies, serum creatinine was measured using a Jaffe method and calibrated to nationally representative estimates as described previously [22]. GFR was estimated using the 4-variable MDRD Study equation [23]. CKD was defined as eGFR <60 ml/min/1.73 m^2^ as described in the GWAS publication that identified the association between common UMOD variants and CKD [6].

### Selection of individuals for sequencing

In the FHS Study, 50 men and 50 women of European ancestry were selected from each extreme of the urinary uromodulin-to-creatinine ratio, for a total of 200 individuals. This design allows for identifying variants in *UMOD* that lead to both extremely high and low concentrations of urinary uromodulin.

In the ARIC Study, 285 individuals of European ancestry were selected based on the presence or absence of CKD as well as on the 8-SNP haplotype identified from GWAS (rs12917707, rs12922822, rs13329952, rs13333226, rs4293393, rs13335818, rs9928936, rs9928757; high CKD-risk haplotype marked by the common C allele at rs12917707) [6]. In total, we sequenced 96 of 320 individuals with CKD and homozygous for the at-risk haplotype, 95 of 2,193 individuals without CKD (defined as eGFR between 80 and 120 ml/min/1.73 m^2^) and homozygous for the at-risk haplotype, and 94 of 101 individuals without CKD and homozygous for the protective haplotype; there were too few individuals to select from the group with CKD and homozygous for the protective haplotype (n = 11). This selection scheme enhanced our ability to identify single common variants that could account for the observed GWAS signal.

### Sequencing and Follow-up Genotyping

Sequencing of the *UMOD* exons and the 4 kb upstream region including putative regulatory elements was conducted for the FHS samples using Sanger sequencing on a ABI 3730 Capillary Sequencer at Beckman Coulter Genomics. Twenty-two amplicons were used to cover the sequenced region on chromosome 16; the coordinates of the sequenced region were 20368293-20367780, 20367984-20367476, 20367640-20367050, 20367305-20366749, 20366892-20366350, 20366480-20365913, 20366084-20365491, 20365633-20365045, 20365258-20364659, 20364911-20364313, 20364453-20363910, 20362361-20361850, 20360710-20360211, 20360359-20359766, 20359909-20359435, 20357762-20357178, 20355642-20355199, 20352794-20352346, 20349081-20348482, 20348413-20347818, 20347024-20346523, and 20344820-20344290. Three out of 63 SNPs were excluded from association analyses by applying Polyphred score threshold 99 or above that corresponds to 97% true positive rate of a SNP calling (http://droog.mbt.washington.edu/poly_doc50.html).

In the ARIC Study, two stranded sequencing was conducted using capillary-based Sanger sequencing (3730xl DNA Analyzer) at the University of Texas Health Science Center at Houston. *UMOD* exons were resequenced using the VariantSEQr platform (16 amplicons), which allows amplification of exons with similar PCR conditions and sequencing with a single universal primer. The *UMOD* upstream region not covered by VariantSEQr was amplified using custom designed primers (one amplicon). The positions of the sequenced regions were 20367823-20367620, 20365191-20363719, 20362316-20361844, 20360580-20359395, 20357704-20357239, 20355525-20355309, 20352722-20352486, 20349045-20348548, 20348307-20347844, 20347060-20346650, and 20344840-20344114 (NC_000016.9 GRCh37.p5 coordinates). Variants were identified using the Lasergene v8 SeqMan assembler, and SNP traces visually verified for all subjects at putative SNP sites. One subject was excluded due to >50% missing data, and one variant was excluded due to a call rate <70%. Sequencing primers for both studies are available upon request.

All non-synonymous coding variants as well as novel associated variants in the putative promoter region were selected for follow-up genotyping in the respective discovery study. In FHS, the five non-synonymous coding variants as well as the upstream variant g.20364263C>T were genotyped among n = 7,567 with GFR and n = 2,522 with available urinary uromodulin data. In ARIC, the non-synonymous coding variant V458L, the upstream variant rs28362063 and rs111699931 in the 3′ UTR were followed up among 14,635 individuals. **Figure S1** shows a flow chart of the study design.

Follow-up genotyping of selected variants in both ARIC and FHS was conducted using Taqman assays at the University of Texas Health Science Center at Houston. Concordance of a set of 62 samples genotyped in duplicate was 100%. Genotyping quality control metrics for the variants are provided in **Table S1**. Genotyping quality was high, and all rare variants identified through sequencing were confirmed by genotyping, even in cases where just one copy of the alternate allele had been observed in the sequence data.

### Functional studies of variant effect on uromodulin polymerization and trafficking

HA-tagged uromodulin isoforms were expressed from pcDNA 3.1(+) (Invitrogen, Carlsbad, CA) as previously described [24]. The V458L variant was obtained by using the Quickchange mutagenesis kit (Stratagene, La Jolla, CA) following the manufacturer's instructions (primers available upon request). The construct was fully sequenced to verify the mutagenesis. The protocols for the generation of stably transfected cells, western blot and immunofluorescence analyses have been previously described [24]. Antibodies used were mouse monoclonal anti-HA (Covance Research Products, Princeton, NJ), mouse monoclonal anti-alpha tubulin (Santa Cruz Biotechnology, Santa Cruz, CA), and rabbit polyclonal anti-calnexin (Sigma-Aldrich, Saint Louis, MO).

### Statistical analyses

Study sample characteristics were compared among the groups selected for sequencing using t-test and ANOVA for continuous variables and chi-square test for categorical variables.

Associations with the urinary uromodulin-to-creatinine ratio, GFR and CKD in the sequenced samples were evaluated using linear and logistic regression models assuming an additive genetic model. In ARIC, p-values for variants with MAF<0.05 were obtained using adaptive permutation. Both the urinary uromodulin-to-creatinine ratio and eGFR were analyzed using a natural logarithmic transformation due to their skewed nature. Throughout this manuscript, “THP” is used to refer to the ln(urinary uromodulin to creatinine ratio).

For the follow-up analyses of the genotyped variants, linear regression was used to evaluate the associations between genotype and THP and eGFR levels. In both studies, analyses were adjusted for age, sex, cohort status (FHS only) and study center (ARIC only), and an additive genetic model was used. In FHS, empirical p-values from testing simulated genotypes according to family relationships with observed phenotypes were reported to account for phenotypic correlation due to family relationship.

Cumulative effects of multiple rare variants on THP concentrations adjusted for age, sex and cohort status were tested using the sequence kernel association test (SKAT) [25].

The expected proportion of variants discovered was calculated using the method in Li and Leal 2009 [26]. Post-hoc power was calculated for detecting significant associations for variants brought forward to follow-up genotyping.

## Results

### Variants Identified through Sequencing

Characteristics of the individuals selected for sequencing are presented by study and group of high or low selection criterion (THP, CKD) in **Table 1**.

**Table 1:** Characteristics of the study samples used for sequencing.

	Framingham Heart Study			ARIC Study			
	Low THP levels (n = 100)	High THP levels (n = 100)	P-value	Cases (n = 96)	Controls low risk hap (n = 94)	Controls high risk hap (n = 95)	P-value
**Age, years**	55.8(9.0)	59.0(9.7)	0.017	66. (6)	64 (6)	63 (5)	0.0004
**Uromodulin (ug/ml)**	0.23(0.30)	34.6(26.1)	<.0001	NA	NA	NA	NA
**Uromodulin/U-creatinine** **ratio**	0.0018(0.0019)	0.41(0.23)	<.0001	NA	NA	NA	NA
**% female (n)**	50.0*(50)	50.0*(50)	1	60.4 (58)	47.9 (45)	51.6 (49)	0.2
**% HTN (n)**	42.0(42)	34.0(34)	0.25	59.4 (57)	35.1 (33)	29.5 (28)	<0.001
**% DM (n)**	10.0(10)	3.0(3)	0.045	13.5 (13)	20.4 (19)	10.5 (10)	0.15
**BMI, kg/m2**	29.8(6.2)	27.1(4.3)	0.0003	28.7 (4.6)	28.1 (4.9)	27.2 (4.6)	0.11
**GFR, ml/min/1.73 m^2^**	94.7(24.0)	90.0(25.4)	0.18	54.3 (7.2)	85.5 (12.0)	86.7 (14.0)	<0.001
**UACR (mg/g)**	2.5(2.8)	30.8(96.8)	0.004	5.9 (5.6)	5.8 (5.1)	4.6 (3.9)	0.1

For continuous variables, the standard deviation is shown in parentheses; for dichotomous variables, this is the n.

* matched by design. Abbreviations: HTN: hypertension; DM: diabetes mellitus; BMI: body mass index; GFR: glomerular filtration rate; UACR: urinary albumin to creatinine ratio. P-values for differences across the three groups in ARIC are obtained by ANOVA or chi^2^ tests.

**Figure 1:**
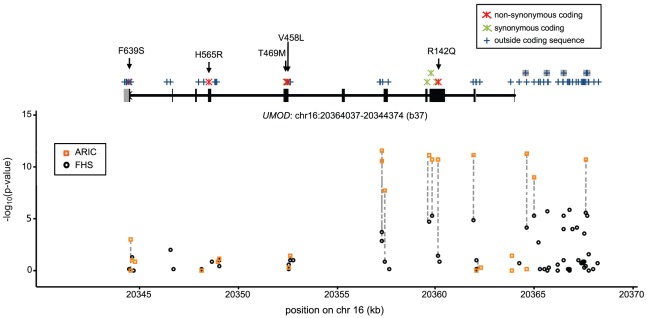
Structure of the *UMOD* gene and localization of the variants identified through sequencing as well as their association with phenotypes. In the *UMOD* gene, exons are indicated as black boxes and 3′ and 5′ UTR as gray boxes. Identified variants are shown as a rug mark on top of the gene, common variants that comprised the initial GWAS signal are shown as the upper part of the rug mark and are highlighted in gray if non-exonic. Non-synonymous variants are indicated. In the lower part, variants that are identified in both studies are connected with a gray dotted line, with their −log_10_(p-value) for association with eGFR (ARIC) and THP (FHS) shown on the ordinate.

**Table 2** provides an overview of the variants identified through sequencing. In the 200 individuals from the FHS Study, 63 variants in *UMOD* exons as well as the entire 4 kb upstream region were identified. In the 285 sequenced samples from the ARIC Study, 24 variants in *UMOD* exons, upstream and conserved 5′ regions were identified. Most of the additional variants found in FHS were due to the larger sequenced upstream region. In both studies, more than half of the identified variants were of low frequency (MAF<0.05), and about 25% of variants in both studies are not contained in the current publicly available databases (dbSNP Build 134, release August 15, 2011). There were 4 synonymous coding variants identified in both studies, and 5 non-synonymous coding variants, one found in both studies and 4 exclusively in the FHS Study (**Table 2**). **Figure 1** shows an overview of all identified variants and their localizations with respect to the *UMOD* gene. All variants identified through sequencing as well as their associations with eGFR (ARIC) and THP (FHS) are listed in **Tables S2 and S3**. Although different primary phenotypes were analyzed in the two studies, the same variants showed significant associations with eGFR (ARIC) and THP (FHS) (**Figure 1**). Of the 17 variants present in both studies, all 11 variants with MAF>0.05 showed associations with either higher THP in FHS and lower GFR in ARIC, or with lower THP in FHS and higher GFR in ARIC, consistent with earlier GWAS findings. This was also the case for 4 of the 6 rare variants found in both studies (compare **Tables S2 and S3**).

**Table 2:** Summary of Variants Identified via sequencing in each study.

	FHS	ARIC
n (individuals)	200	285
% missing data	2.8	7.1
# variants (common/rare*)	63 (22/41)	24 (11/13)
# variants with rs number	46 (22/24)	18 (11/7)
# synonymous coding variants	4 (3/1)	4 (3/1)
# non-synonymous coding variants	5 (0/5)	1 (0/1)

* rare in this context is defined as MAF<0.05. Known variants are based on dbSNP134, HRG37.2. Of all variants identified in total, 17 were identified in both studies (7 rare/10 common [referring to FHS minor allele frequency]). Of these, four variants were synonymous coding (3 common, 1 rare) and one non-synonymous coding.

### Follow-up genotyping in population-based studies

**Figure 2:**
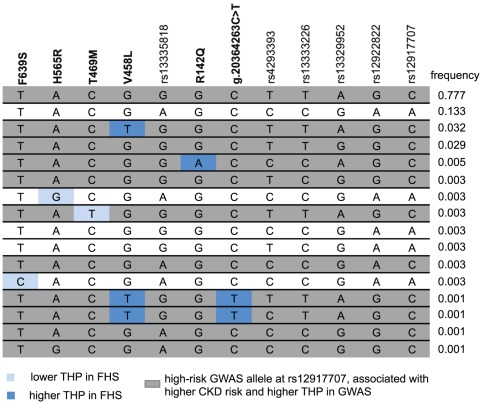
Haplotypes at the *UMOD* locus in the 200 sequenced FHS participants. Haplotypes are composed of the 6 rare variants detected in this study (bold) and 6 common variants tagging the GWAS signal. Frequencies of each haplotype are provided to the right, and the haplotypes containing the high CKD-risk allele at rs12917707 (C) are shaded in gray. Rare alleles associated in this study with lower THP concentrations are shown in light blue, and rare alleles associated with higher THP concentrations are shown in dark blue.

**Table 3** provides an overview of the variants selected for follow-up genotyping, including any resulting amino acid substitutions and information about functional prediction. Both predicition softwares Polyphen2 and SIFT classified the V458L, T469M, and H565R as damaging, whereas R142Q, located in the third epidermal growth factor domain of uromodulin, and F639S were classified as benign. In FHS, all non-synonymous coding variants, as well as any novel rare eGFR- or THP-associated variants in the upstream region were followed up (total of 6 variants). **Figure 2** shows the haplotypes formed by these 6 rare variants as well as 6 common variants that are tag SNPs for the original GWAS signal among the 200 sequenced FHS participants, in order to assess whether rare alleles leading to non-synonymous amino acid changes always occur on the same haplotypic background. Although the rare allele leading to the V458L change that was associated with higher THP concentrations was always found on the high-risk haplotype identified in GWAS, there were also rare alleles associated with lower THP identified on the high-risk GWAS haplotype (T469M). In ARIC, all non-synonymous coding variants as well as novel common variants in the conserved 3′ or 5′ region of the *UMOD* gene or falling into a predicted transcription factor binding site were followed up (total of 3 variants). Of the five non-synonymous coding variants typed between the two studies, four had already been deposited in dbSNP 134 [27] or identified through other sequencing efforts [28].

**Table 3:** information about variants selected for follow-up genotyping.

variant	rs28362063	g.20364263C>T	R142Q	V458L (rs55772253)	T469M (rs143583842)	H565R	F639S (rs145165861)	rs111699931
**bp**	20365012	20364263	20360198	20352618	20352584	20348659	20344643	20344532
**localization**	upstream (−2953 bp*)	upstream (−2204 bp*)	exon 3	exon 7	exon 7	exon 8	exon 11	3′ UTR
**alleles (major/minor)**	A/G	C/T	G/A	G/T	C/T	A/G	T/C	C/T
**MAF in sequenced sample**	0.33**	0.003	0.005	0.035	0.003	0.003	0.003	0.08**
**MAF in genotyped sample**	0.18**	0.0001	0.0023	0.026	0.0016	0.0004	0.0004	0.0375**
**consequence**	in transcription factor (CEBPB) binding site	putative promoter region	Arg –>Gln	Val –>Leu	Thr –>Met	His –>Arg	Phe –>Ser	3′UTR
**functional prediction Polyphen2**	NA	NA	benign; score 0.15 (sens 0.93, spec 0.86)	probably damaging; score 0.99 (sens 0.68, spec 0.97)	probably damaging; score 1.0 (sens 0, spec 1)	probably damaging; score 1.0 (sens 0.14, spec 0.99)	unknown	NA
**functional prediction SIFT**	NA	NA	tolerated	damaging	damaging	damaging	tolerated	NA

* numbered according to the UMOD start codon;

** number provided for ARIC, otherwise for the FHS sample; alleles are on the – strand from which UMOD is transcribed; SIFT was run on dbSNP version 132; Polyphen2 was based on the UniProtKB/UniRef100 Release 2011_04; 1000 Genome information is based on the August 2010 release.

### Association of genotyped variants with THP and eGFR in population-based studies

The association between eGFR and each variant in the follow-up samples are provided in **Tables 4** **(FHS) and 5 (ARIC)**. Only V458L was nominally associated; each copy of the minor T allele was associated with lower eGFR (p = 0.046) in ARIC. Conversely, the association with higher eGFR in FHS was not significant (p = 0.07). In the ARIC Study, V458L was not able to account for the GWAS signal. The r^2^ and D′ between the V458L and rs12917707, tagging the original GWAS signal, in the entire ARIC European-ancestry sample was 0.002 and 0.735, respectively. Moreover, the inclusion of V458L in a regression model that already contained rs12917707 did not explain additional variance of eGFR (2.7% in model with rs12917707 alone and 2.8% in a model with rs12917707 and V458L) nor did it appreciably change each SNP's beta estimates and p values. In FHS, the minor allele of g.20364263 was associated with higher levels of eGFR (p = 7×10^−3^), but only 2 copies of the minor allele were present in 7,520 FHS individuals.

**Table 4:** Associations between genotyped variants identified from sequencing brought forward to the entire FHS sample.

		20364263	20360198	rs55772253	rs143583842	20348659	rs145165861
	Phenotype	C/T	R142Q	V458L	T469M	H565R	F639S
Alleles (major/minor)	log(eGFR)	C/T	G/A	G/T	C/T	A/G	T/C
effect, per allele		0.53	−0.027	0.023	0.0047	−0.0020	0.011
se		0.16	0.038	0.011	0.050	0.091	0.091
p-value		6.8E-03	0.53	0.07	0.93	0.98	0.90
n		7520	7534	7520	7527	7529	7523

Alleles (major/minor)	log(urinary uromodulin- to-creatinine ratio)	C/T	G/A	G/T	C/T	A/G	T/C
effect, per allele		2.57	0.28	0.32	−0.50	−2.54	−0.64
se		1.37	0.40	0.12	0.61	0.97	0.97
p-value		0.066	0.49	9.2E-03	0.41	0.023	0.48
n		2492	2496	2490	2491	2496	2495

**Table 5:** Associations between genotyped variants identified from sequencing brought forward to the entire ARIC sample and ln(eGFR).

	rs28362063	rs55772253 (V458L)	rs111699931
**ARIC white**			
Alleles (major/ minor)	A/G	G/T	C/T
effect, per allele	0.015	−0.021	0.002
se	0.004	0.010	0.008
p-value	4.98E-05	0.045	0.77
n	10871	10823	10905
**ARIC black**			
Alleles (major/ minor)	A/G	G/T	C/T
effect, per allele	−0.007	0.047	0.038
se	0.015	0.054	0.039
p-value	0.66	0.39	0.33
n	3853	3812	3860

**Table 4** also shows the association between the genotyped variants and THP in the FHS Study. Significant associations were found between the minor T allele of V458L and higher THP (p = 9*10^−3^), as well as the minor G allele of H565R and lower THP (p = 0.02). When conducting the SKAT burden test among the entire genotyped population to test for the cumulative effects of all rare variants, the test was significant (p = 0.007). Excluding V458L and g.20364263C>T from the burden test, as the first variant has a minor allele frequency of 0.03 and the second variant is not located within the gene, yielded a non-significant p-value (p = 0.29).

In the FHS Study, the previously published GFR-associated variant from GWAS, rs12917707, explained 2.3% of the THP variance. Together, the 6 genotyped rare variants explained only an additional 0.5% of variance. We also examined if any additional sequence SNPs in the *UMOD* promoter region explained additional variation in the presence of the GWAS SNP rs12917707 (p = 4×10^−6^ in the sequenced sample). SNPs that remained nominally significant (p = 0.03) and therefore contributed to explaining additional THP variance after adjusting for rs12917707 were rs115312009, rs147825435, rs76619864, and rs75432530.

### Functional assessment of the V458L variant

In our study, the V458L variant was the only rare variant that showed association with both THP concentrations (FHS) and eGFR (ARIC). Therefore, we evaluated whether the V458L variant would show characteristics typical of variants causing monogenic *UMOD* disease, since patients with rare *UMOD* mutations show lower urinary uromodulin levels, which are caused by incorrect protein folding, trafficking and aggregation. The low frequency variant V458L leads to an amino acid substitution of valine to leucine at position 458 in exon 7 of *UMOD* and is predicted to have a damaging effect on protein function by both PolyPhen-2 [29] and SIFT [30]; three other patient mutations in monogenic *UMOD* diseases have been described in exon 7 [16], [31].

**Figure 3:**
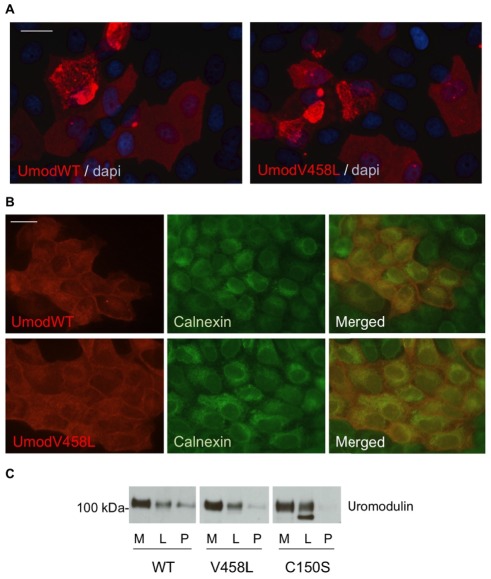
Analysis of trafficking and polymerization of uromodulin variant V458L. Immunofluorescence showing uromodulin distribution in MDCK cells stably expressing HA-tagged wild-type or V458L isoforms. Bar = 20 µm. (**a**) Unpermeabilized cells: both isoforms traffic to the plasma membrane where they assemble into polymeric filaments. (**b**) Permeabilized cells: V458L isoform is not enriched in the ER compared to the wild type. Calnexin is shown as an ER marker. (**c**) Western-blot detection of HA-tagged uromodulin isoforms from the medium (M) and the soluble (L) and unsoluble (P) fractions of lysates from stably transfected MDCK cells. While pathogenic mutant C150S shows enrichment of the ER precursor, the V458L isoform is not different from the wild type one.

In stably transfected MDCK cells the UMOD V458L variant did not show any significant difference relative to wild-type protein. Indeed, it reached the plasma membrane in comparable amount, it assembled into extracellular filaments (**Figure 3A**) and it was not enriched in the ER, as opposed to the patient mutation C150S with a known trafficking defect [32] (**Figure 3B, C**).

## Discussion

In our study of targeted sequencing of coding and conserved portions of the *UMOD* gene region, we did not identify any common non-synonymous coding variant that could account for the GWAS signal by itself. Rare variants were identified and verified, but they were not significantly enriched among individuals at the extremes of THP levels when the entire study population was considered. The V458L variant, which is predicted to have a damaging effect on protein function, showed significant association with eGFR in one but not both studies, association with THP, and no apparent effect in functional studies on protein aggregation or trafficking as observed for monogenic disease-causing variants.

### Interpretation of association results

Several potential reasons exist for our observations. First, there may not be any moderately-to-highly penetrant rare *UMOD* variants associated with the common, complex phenotypes under study. Second, we only focused on sequencing the exons of *UMOD* and the 4 kb upstream region. Even though our GWAS signal was localized to the promoter region upstream of *UMOD*, it is possible that the causal, possibly non-SNP, variant localizes to the non-coding regions of the gene or beyond the 4 kb upstream region. Third, we sequenced approximately 200–300 individuals from each study, and thus may have missed other rare variants. For a sequenced sample size of 94–100 in each of the case/control groups, we expect to discover 85% of all variants with MAF>1% and 64% of all variants with MAF>0.5%. The percentage of trait variance explained by the rare SNPs ranged from 5*10^−8^ for H565R for eGFR in FHS to 0.27% for V458L for THP concentrations in FHS (median 0.042%). At a nominal alpha level of 0.05, to achieve 80% power, the required sample size is 2,900 for detecting a SNP explaining 0.27% of the trait variance, and 18,550 for 0.042% of the trait variance. It is therefore possible that we were underpowered to identify robust associations of some of our newly identified rare variants in the general population. Even so, our results indicate that none of the identified rare SNPs had a large effect on the phenotypes. Finally, in terms of the unchanged functional effects of the V458L variant, the variant may exert its effects on THP concentrations and possibly renal function through a different mechanism than the one known for monogenic disease and that is not studied in the present study.

### In the context of monogenic uromodulin-associated kidney diseases

The rare non-synonymous coding variants R142Q, T469M, H565R, and F639S in *UMOD* identified in the FHS Study had not previously been described in reports of patients with rare autosomal-dominant uromodulin-associated diseases. Recently, the largest description of kidney disease patients with *UMOD* mutations to date was published [31]. In this report, the authors also identified the T469M variant, which they classified as a patient variant defined as a missense mutation affecting a highly conserved nucleotide and absent in 300 control chromosomes. In our study, we observed 25 carriers of one copy of the T469M minor allele out of a total of 7,919 genotyped FHS participants. The Mendelian forms of uromodulin-associated disease exhibit an autosomal-dominant mode of inheritance, and the median renal survival in individuals with Mendelian forms of disease is estimated at 54 years [31]. As none of the carriers of the T469M minor allele had end-stage renal disease, our results suggest that either T469M may not be a causal variant for Mendelian forms of uromodulin-associated disease, or it is a mild one with a modest effect on renal survival.

### Comparison to other similar studies

Several previous studies have re-sequenced candidate genes identified through GWAS in order to identify novel rare genetic variants with potentially stronger associations with the phenotype than observed for the trait-associated variants in GWAS. More successful recent examples in the area of common, complex diseases include uric acid and gout [33] and inflammatory bowel disease. Using 350 cases of Crohn's disease and controls, investigators were able to identify multiple independent rare variants in 7 genes, some with large effect sizes [34]. Similar to the present study, the variants identified did not account for the original GWAS signal. However, less success has been observed for many other common complex diseases, including fine-mapping of the 9p21 region in association with type 2 diabetes and coronary artery disease [35], and type 1 diabetes [36]. There are currently many ongoing sequencing efforts that are focused on sequencing the exons of candidate genes, as well as whole-exome sequencing. The relative yield of this approach, as compared to whole genome sequencing and assessment of structural variation, remains unknown.

### Conclusion

Multiple novel rare non-synonymous coding variants in the *UMOD* region were identified, but none were consistently associated with eGFR. Only V458L had modest association with THP levels in the general population and thus could not account for the observed GWAS signal.

## Supporting Information

Figure S1 **Flow chart of the study design.**
(TIF)

Table S1 **Genotyping quality metrics.**
(DOC)

Table S2 **All Variants identified in the ARIC Study.**
(DOC)

Table S3 **All Variants identified in the Framingham Heart Study.**
(DOC)
