# Supplementary figures and images for: Association of Estimated Glomerular Filtration Rate and Urinary Uromodulin Concentrations with Rare Variants Identified by UMOD Gene Region Sequencing
Source: PLoS One. 2012 May 31;7(5):e38311. doi: 10.1371/journal.pone.0038311 (PMC3365030; doi:10.1371/journal.pone.0038311)

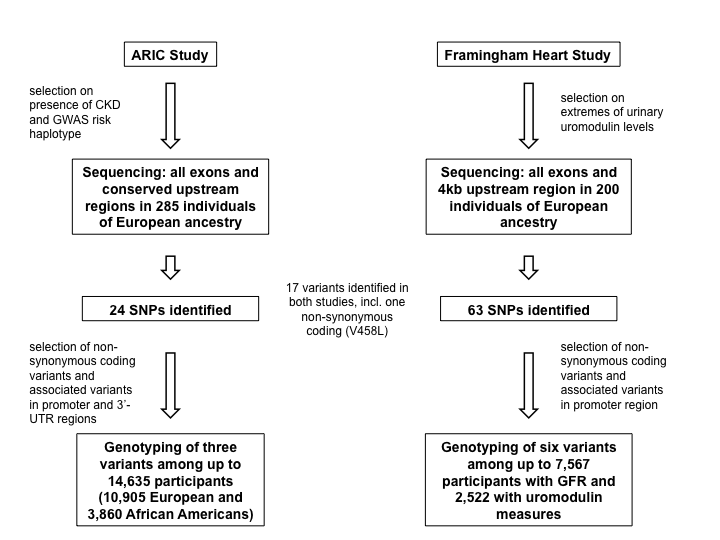

Supplement: Figure S1 — Flow chart of the study design. (TIF) [file pone.0038311.s001.tif]
