# Supplementary material for: Association of Estimated Glomerular Filtration Rate and Urinary Uromodulin Concentrations with Rare Variants Identified by UMOD Gene Region Sequencing
Source: PLoS One. 2012 May 31;7(5):e38311. doi: 10.1371/journal.pone.0038311 (PMC3365030; doi:10.1371/journal.pone.0038311)
Supplement: Table S1 — Genotyping quality metrics. (DOC) [file pone.0038311.s002.doc]

**Table S1: Genotyping quality metrics**

| variant | rs28362063 | g.20364263C>T | R142Q | V458L | T469M | H565R | F639S | rs111699931 |
| --- | --- | --- | --- | --- | --- | --- | --- | --- |
| major allele | A | C | G | G | C | A | T | C |
| minor allele | G | T | A | T | T | G | C | T |
| MAF | 0.18 | 0.0001 | 0.002 | 0.03 | 0.002 | 0.0004 | 0.0004 | 0.04 |
| minor allele count | 3930 | 2 | 36 | 411 | 25 | 6 | 6 | 823 |
| p-HWE | 0.50 | 1 | 1 | 0.38 | 1 | 1 | 1 | 0.08 |
| n | 10945 | 7913 | 7925 | 7911 | 7919 | 7923 | 7916 | 10979 |
| call rate | 0.98 | 0.99 | 0.99 | 0.99 | 0.99 | 0.99 | 0.99 | 0.98 |

Values for rs28362063 and rs111699931 are from the genotyped ARIC samples, the rest of the variants from the genotyped FHS samples
