# Supplementary material for: Association of Estimated Glomerular Filtration Rate and Urinary Uromodulin Concentrations with Rare Variants Identified by UMOD Gene Region Sequencing
Source: PLoS One. 2012 May 31;7(5):e38311. doi: 10.1371/journal.pone.0038311 (PMC3365030; doi:10.1371/journal.pone.0038311)
Supplement: Table S2 — All Variants identified in the ARIC Study. (DOC) [file pone.0038311.s003.doc]

**Table S2: All Variants identified in the ARIC Study**

|  |  |  |  |  |  |  | **ln(eGFR)** | | | |
| --- | --- | --- | --- | --- | --- | --- | --- | --- | --- | --- |
| **pos** | **Gene Position** | **rs ID** | **gene region and variant function** | **ARIC alleles (major / minor)** | **MAF ARIC** | **ARIC % missing** | **beta** | **se** | **p** | **n perm** |
| 20367645 | -5586 | rs12922822 | Promoter | G / A | 0.332 | 0.28 | 0.110 | 0.016 | 2.0E-11 | NA |
| 20365012 | -2953 | rs28362063 | Promoter | A / G | 0.328 | 0.08 | 0.102 | 0.016 | 9.1E-10 | NA |
| 20364638 | -2579 |  | Promoter | C / T | 0.002 | 0.12 | 0.113 | 0.267 | 0.67 | 11 |
| 20364588 | -2529 | rs4293393 | Promoter | T / C | 0.328 | 0.12 | 0.111 | 0.015 | 4.7E-12 | NA |
| 20363839 | -1780 | rs76563024 | Intron 1 | T / C | 0.015 | 0.12 | -0.009 | 0.094 | 0.78 | 8 |
| 20363838 | -1779 |  | Intron 1 | C / T | 0.002 | 0.10 | -0.488 | 0.260 | 0.03 | 613 |
| 20362285 | -226 | rs117738531 | Intron 1 | T / C | 0.005 | 0.10 | -0.139 | 0.154 | 0.41 | 31 |
| 20362106 | -47 | rs75645968 | 5' UTR | T / C | 0.014 | 0.06 | 0.003 | 0.095 | 1 | 6 |
| 20361950 | 110 | rs36060036 | Intron 2 | G / A | 0.306 | 0.05 | 0.118 | 0.016 | 6.1E-12 | NA |
| 20360101 | 1959 | rs7193058 | Exon 3, synonymous coding | T / C | 0.419 | 0.05 | 0.116 | 0.016 | 1.6E-11 | NA |
| 20359831 | 2229 | rs13335818 | Exon 3, synonymous coding | G / A | 0.326 | 0.05 | 0.110 | 0.016 | 1.6E-11 | NA |
| 20359633 | 2427 | rs28544423 | Exon 4, synonymous coding | G / A | 0.329 | 0.05 | 0.112 | 0.016 | 6.1E-12 | NA |
| 20357398 | 4662 | rs4506906 | Intron 5 | T / C | 0.498 | 0.04 | 0.097 | 0.017 | 2.0E-08 | NA |
| 20357281 | 4779 | rs12934455 | Intron 5 | G / A | 0.299 | 0.04 | 0.113 | 0.016 | 2.7E-11 | NA |
| 20357255 | 4805 | rs12934320 | Intron 5 | G / A | 0.335 | 0.12 | 0.112 | 0.015 | 2.4E-12 | NA |
| 20352618 | 9442 | rs55772253 | Exon 7, non-synonymous coding | G / T | 0.015 | 0.02 | -0.195 | 0.094 | 0.03 | 628 |
| 20352532 | 9528 | rs141800038 | Exon 7, synonymous coding | C / T | 0.009 | 0.01 | -0.063 | 0.119 | 0.46 | 27 |
| 20348995 | 13065 | rs72776658 | Intron 7 | G / A | 0.018 | 0.02 | -0.137 | 0.082 | 0.07 | 277 |
| 20348957 | 13103 |  | Intron 7 | T / A | 0.002 | 0.06 | 0.383 | 0.270 | 0.12 | 159 |
| 20348137 | 13923 | rs146004778 | Intron 8 | C / T | 0.004 | 0.06 | -0.151 | 0.188 | 0.78 | 8 |
| 20344735 | 17325 |  | Intron 10 | C / T | 0.004 | 0.00 | -0.287 | 0.188 | 0.14 | 133 |
| 20344628 | 17432 |  | 3' UTR | C / T | 0.002 | 0.03 | 0.407 | 0.265 | 0.10 | 191 |
| 20344532 | 17528 | rs111699931 | 3' UTR | C / T | 0.061 | 0.06 | 0.156 | 0.046 | 8.1E-04 | NA |
| 20344496 | 17564 |  | 3' UTR | C / T | 0.002 | 0.05 | 0.092 | 0.264 | 0.86 | 6 |

The gene position is with reference to the UMOD ATG start codon; gene coordinates are those of NC_000016.9 Reference GRCh37.p5 Primary Assembly (20344373..20364037). The corresponding RefSeqGene is NG_008151.1. Alleles are provided on the – strand
