# Supplementary material for: Association of Estimated Glomerular Filtration Rate and Urinary Uromodulin Concentrations with Rare Variants Identified by UMOD Gene Region Sequencing
Source: PLoS One. 2012 May 31;7(5):e38311. doi: 10.1371/journal.pone.0038311 (PMC3365030; doi:10.1371/journal.pone.0038311)
Supplement: Table S3 — All Variants identified in the Framingham Heart Study. (DOC) [file pone.0038311.s004.doc]

**Table S3: All Variants identified in the Framingham Heart Study**

|  |  |  |  |  |  |  | **ln(urinary uromodulin-to-creatinine ratio)** | | | **ln(eGFR)** | | |
| --- | --- | --- | --- | --- | --- | --- | --- | --- | --- | --- | --- | --- |
| **pos b37** | **Gene Position** | **rs ID db SNP 134** | **gene region and variant function** | **FHS alleles (major / minor)** | **MAF FHS** | **FHS % missing** | **beta** | **se** | **p** | **beta** | **se** | **p** |
| 20368254 | -6195 | rs75432530 | Promoter | G/A | 0.0050 | 0.5 | 3.19 | 2.09 | 0.16 | -0.225 | 0.169 | 0.18 |
| 20368027 | -5968 |  | Promoter | C/T | 0.0050 | 0 | -0.67 | 2.11 | 0.63 | -0.144 | 0.170 | 0.38 |
| 20367763 | -5704 | rs147588693 | Promoter | C/T | 0.0025 | 1 | -2.27 | 2.99 | 0.77 | 0.305 | 0.241 | 0.18 |
| 20367741 | -5682 | rs80159442 | Promoter | C/T | 0.0025 | 0.5 | -4.50 | 2.97 | 0.02 | 0.312 | 0.241 | 0.17 |
| 20367690 | -5631 | rs12917707 | Promoter | C/A | 0.1432 | 0.5 | -1.79 | 0.38 | 4.3E-06 | 0.016 | 0.032 | 0.62 |
| 20367645 | -5586 | rs12922822 | Promoter | G/A | 0.1457 | 0.5 | -1.83 | 0.38 | 2.5E-06 | 0.022 | 0.032 | 0.49 |
| 20367617 | -5558 |  | Promoter | T/C | 0.0025 | 0.5 | -3.18 | 2.97 | 0.35 | -0.226 | 0.241 | 0.34 |
| 20367550 | -5491 | rs28688991 | Promoter | G/A | 0.1818 | 1 | -1.37 | 0.36 | 2.2E-04 | 0.042 | 0.030 | 0.17 |
| 20367516 | -5457 | rs115312009 | Promoter | G/A | 0.0051 | 1 | 3.20 | 2.10 | 0.14 | -0.225 | 0.170 | 0.18 |
| 20367514 | -5455 |  | Promoter | T/C | 0.0025 | 1 | -3.04 | 2.98 | 0.41 | -0.009 | 0.242 | 0.98 |
| 20367500 | -5441 | rs147825435 | Promoter | A/G | 0.0051 | 1 | 3.20 | 2.10 | 0.14 | -0.225 | 0.170 | 0.18 |
| 20367455 | -5396 | rs76619864 | Promoter | C/T | 0.0051 | 1.5 | 3.19 | 2.10 | 0.15 | -0.225 | 0.170 | 0.18 |
| 20367389 | -5330 |  | Promoter | C/A | 0.0025 | 1.5 | 3.74 | 2.98 | 0.16 | 0.730 | 0.236 | 0.01 |
| 20367239 | -5180 | rs7204342 | Promoter | C/A | 0.0352 | 0.5 | 1.42 | 0.81 | 0.09 | 0.077 | 0.066 | 0.25 |
| 20367130 | -5071 | rs7203642 | Promoter | T/C | 0.1784 | 0.5 | -1.49 | 0.36 | 5.8E-05 | 0.040 | 0.030 | 0.19 |
| 20366949 | -4890 | rs7204775 | Promoter | A/G | 0.1710 | 3.5 | -1.51 | 0.37 | 7.9E-05 | 0.046 | 0.031 | 0.14 |
| 20366810 | -4751 | rs9933330 | Promoter | G/A | 0.1482 | 0.5 | -1.87 | 0.37 | 1.3E-06 | 0.018 | 0.032 | 0.57 |
| 20366779 | -4720 | rs71384447 | Promoter | C/A | 0.0225 | 0 | 0.32 | 1.01 | 0.76 | -0.055 | 0.082 | 0.50 |
| 20366765 | -4706 | rs149977864 | Promoter | T/C | 0.0027 | 8.5 | 2.71 | 2.95 | 0.56 | -0.372 | 0.240 | 0.12 |
| 20366754 | -4695 | rs139558165 | Promoter | G/T | 0.0028 | 10.5 | 2.66 | 2.94 | 0.60 | -0.375 | 0.240 | 0.12 |
| 20366507 | -4448 | rs13329952 | Promoter | A/G | 0.1769 | 2.5 | -1.50 | 0.37 | 7.7E-05 | 0.027 | 0.031 | 0.39 |
| 20366477 | -4418 | rs74842838 | Promoter | T/C | 0.0282 | 2.5 | 1.56 | 0.91 | 0.09 | 0.111 | 0.073 | 0.14 |
| 20366459 | -4400 | rs13334589 | Promoter | T/A | 0.1407 | 0.5 | -1.84 | 0.39 | 4.2E-06 | 0.000 | 0.033 | 1.00 |
| 20366225 | -4166 | rs11862974 | Promoter | G/T | 0.0303 | 1 | 1.06 | 0.88 | 0.24 | 0.130 | 0.071 | 0.07 |
| 20366219 | -4160 | rs76608863 | Promoter | A/G | 0.0076 | 1 | -0.59 | 1.73 | 0.84 | -0.095 | 0.140 | 0.48 |
| 20365726 | -3667 |  | Promoter | G/T | 0.0026 | 2 | 3.05 | 2.97 | 0.40 | 0.399 | 0.237 | 0.10 |
| 20365697 | -3638 | rs12708631 | Promoter | T/A | 0.0722 | 3 | 0.06 | 0.59 | 0.93 | -0.001 | 0.047 | 0.99 |
| 20365654 | -3595 | rs13333226 | Promoter | T/C | 0.1563 | 4 | -1.83 | 0.37 | 2.0E-06 | 0.018 | 0.032 | 0.57 |
| 20365498 | -3439 | rs141963187 | Promoter | A/G | 0.0102 | 1.5 | -0.44 | 1.50 | 0.72 | 0.117 | 0.120 | 0.33 |
| 20365324 | -3265 |  | Promoter | G/A | 0.0026 | 2.5 | 2.52 | 2.97 | 0.68 | -0.385 | 0.238 | 0.11 |
| 20365234 | -3175 | rs4997081 | Promoter | C/G | 0.1769 | 2.5 | -1.21 | 0.38 | 1.5E-03 | 0.019 | 0.031 | 0.54 |
| 20365012 | -2953 | rs28362063 | Promoter | A/G | 0.1371 | 7 | -1.89 | 0.40 | 5.2E-06 | -0.015 | 0.034 | 0.66 |
| 20364588 | -2529 | rs4293393 | Promoter | T/C | 0.1462 | 2.5 | -1.60 | 0.39 | 6.2E-05 | -0.001 | 0.033 | 0.97 |
| 20364263 | -2204 |  | Promoter | C/T | 0.0025 | 0.5 | 3.60 | 2.96 | 0.19 | 0.071 | 0.241 | 0.72 |
| 20362106 | -47 | rs75645968 | Intron 1 | T/C | 0.0276 | 0.5 | 1.59 | 0.91 | 0.08 | 0.110 | 0.074 | 0.14 |
| 20362090 | -31 |  | 5' UTR | T/C | 0.0025 | 0.5 | 2.68 | 2.97 | 0.59 | -0.605 | 0.237 | 0.02 |
| 20361950 | 110 | rs36060036 | intron 2 | G/A | 0.1357 | 0.5 | -1.79 | 0.40 | 1.3E-05 | 0.000 | 0.034 | 1.00 |
| 20360198 | 1862 |  | Exon 3, nonsynonymous coding | G/A | 0.0051 | 1 | 3.19 | 2.08 | 0.14 | -0.224 | 0.170 | 0.19 |
| 20360101 | 1959 | rs7193058 | Exon 3, synonymous coding | T/C | 0.2525 | 1 | -0.68 | 0.31 | 0.03 | 0.036 | 0.026 | 0.17 |
| 20359831 | 2229 | rs13335818 | Exon 3, synonymous coding | G/A | 0.1432 | 0.5 | -1.79 | 0.38 | 4.4E-06 | 0.022 | 0.032 | 0.49 |
| 20359633 | 2427 | rs28544423 | Exon 4, synonymous coding | G/A | 0.1475 | 0 | -1.66 | 0.38 | 1.8E-05 | 0.014 | 0.032 | 0.66 |
| 20357678 | 4382 |  | Intron 4 | A/C | 0.0026 | 5 | 2.56 | 2.95 | 0.66 | -0.219 | 0.239 | 0.35 |
| 20357398 | 4662 | rs4506906 | Intron 5 | T/C | 0.3807 | 1.5 | -0.48 | 0.30 | 0.11 | 0.037 | 0.024 | 0.12 |
| 20357281 | 4779 | rs12934455 | Intron 5 | G/A | 0.1236 | 13 | -1.68 | 0.44 | 1.7E-04 | 0.004 | 0.037 | 0.91 |
| 20357255 | 4805 | rs12934320 | Intron 5 | G/A | 0.1307 | 23.5 | -1.51 | 0.46 | 1.2E-03 | 0.018 | 0.038 | 0.64 |
| 20352756 | 9304 | rs9646256 | Intron 6 | C/T | 0.3878 | 2 | -0.50 | 0.30 | 0.10 | 0.024 | 0.024 | 0.31 |
| 20352618 | 9442 | rs55772253 | Exon 7, nonsynonymous coding | G/T | 0.0352 | 0.5 | 1.42 | 0.81 | 0.08 | 0.023 | 0.066 | 0.73 |
| 20352584 | 9476 | rs143583842 | Exon 7, nonsynonymous coding | C/T | 0.0025 | 0.5 | -3.44 | 2.96 | 0.24 | 0.057 | 0.240 | 0.78 |
| 20352532 | 9528 | rs141800038 | Exon 7, synonymous coding | C/T | 0.0101 | 0.5 | 0.67 | 1.50 | 0.66 | -0.169 | 0.121 | 0.16 |
| 20349054 | 13006 | rs72776659 | Intron 7 | C/G | 0.0891 | 13 | 0.47 | 0.53 | 0.37 | -0.047 | 0.043 | 0.27 |
| 20348995 | 13065 | rs72776658 | Intron 7 | G/A | 0.0357 | 2 | 1.39 | 0.81 | 0.09 | 0.019 | 0.066 | 0.78 |
| 20348989 | 13071 |  | Intron 7 | T/C | 0.0026 | 2 | -3.87 | 2.96 | 0.10 | 0.136 | 0.238 | 0.48 |
| 20348659 | 13401 |  | Exon 8, nonsynonymous coding | A/G | 0.0026 | 2.5 | -3.83 | 2.96 | 0.12 | 0.243 | 0.235 | 0.29 |
| 20348137 | 13923 | rs146004778 | Intron 8 | C/T | 0.0025 | 0.5 | 2.61 | 2.97 | 0.63 | 0.342 | 0.239 | 0.14 |
| 20346719 | 15341 |  | Intron 10 | C/T | 0.0025 | 1 | 2.65 | 2.97 | 0.61 | 0.233 | 0.241 | 0.31 |
| 20346545 | 15515 | rs9935075 | Intron 10 | A/G | 0.0127 | 1.5 | -3.40 | 1.32 | 0.01 | 0.243 | 0.108 | 0.03 |
| 20344643 | 17417 | rs145165861 | Exon 11, nonsynonymous coding | T/C | 0.0025 | 0 | -0.98 | 2.97 | 0.98 | -0.097 | 0.240 | 0.61 |
| 20344628 | 17432 |  | UTR3 | C/T | 0.0025 | 0 | 4.23 | 2.96 | 0.04 | -0.367 | 0.239 | 0.12 |
| 20344532 | 17528 | rs111699931 | UTR3 | C/T | 0.0425 | 0 | -0.19 | 0.71 | 0.78 | -0.035 | 0.058 | 0.54 |
| 20344421 | 17639 |  | UTR3 | G/A | 0.0025 | 0 | 2.59 | 2.96 | 0.64 | -0.051 | 0.240 | 0.81 |

positions are provided with respect to the human reference sequence build 37; the SNP numbering relative to gene position is according to the UMOD ATG start codon; alleles are provided on the – strand
